# LiDAR-Based Sensor Fusion SLAM and Localization for Autonomous Driving Vehicles in Complex Scenarios

**DOI:** 10.3390/jimaging9020052

**Published:** 2023-02-20

**Authors:** Kai Dai, Bohua Sun, Guanpu Wu, Shuai Zhao, Fangwu Ma, Yufei Zhang, Jian Wu

**Affiliations:** 1 State Key Laboratory of Automotive Simulation and Control, Jilin University, Changchun 130025, China; 2 Automotive Data Center, CATARC, Tianjin 300000, China

**Keywords:** LiDAR SLAM, autonomous vehicle, localization, multi-sensor fusion

## Abstract

LiDAR-based simultaneous localization and mapping (SLAM) and online localization methods are widely used in autonomous driving, and are key parts of intelligent vehicles. However, current SLAM algorithms have limitations in map drift and localization algorithms based on a single sensor have poor adaptability to complex scenarios. A SLAM and online localization method based on multi-sensor fusion is proposed and integrated into a general framework in this paper. In the mapping process, constraints consisting of normal distributions transform (NDT) registration, loop closure detection and real time kinematic (RTK) global navigation satellite system (GNSS) position for the front-end and the pose graph optimization algorithm for the back-end, which are applied to achieve an optimized map without drift. In the localization process, the error state Kalman filter (ESKF) fuses LiDAR-based localization position and vehicle states to realize more robust and precise localization. The open-source KITTI dataset and field tests are used to test the proposed method. The method effectiveness shown in the test results achieves 5–10 cm mapping accuracy and 20–30 cm localization accuracy, and it realizes online autonomous driving in complex scenarios.

## 1. Introduction

With the development of intelligent and connected vehicle technology, the intelligent transportation system with autonomous driving passenger cars, commercial vehicles and taxis has undergone tremendous changes in the perception of the complex scenarios. Vehicle localization is the key issue that should be solved in autonomous driving and how to realize high-precise vehicle localization under the condition of unavailable satellites or unstructured roads is one of the technical problems to be solved urgently. The localization technique based on the vision [1] and satellites observations can achieve centimeter-level localization but heavily rely on satellite signals, traffic signs, and initialization [2]. LiDAR-based localization techniques are largely invariant to illumination and satellite signal changes. Therefore, high precision maps with denser point clouds are required, and the map-based multi-sensor fusion localization should be widely used to cover different driving conditions [3].

LiDAR SLAM is widely used in the construction of 3D point cloud maps [4]. The architecture of a simultaneous localization and mapping (SLAM) system consists of the front-end and the back-end. The front-end seeks to interpret the sensor data to obtain constraints as the basis for optimization approaches, such as point cloud registration, loop closure detection, or Global Navigation Satellite System (GNSS) pose. The back-end focuses on computing the best map result based on optimization techniques with the given constraints [5]. Many registration methods have been proposed for the front-end, such as the iterative closest point (ICP) [6], normal distribution transformation (NDT) [7], and feature-based [8]. However, typical registration methods suffer from drift in large-scale tests, due to the poor performance in the loop closure detection and the position correction with absolute measurements. The back-end optimization process can reduce the drift based on the typical back-end algorithms, such as the early-used extended Kalman filter (EKF) [9] or the current commonly pose graph optimization [10]. Besides the accuracy and efficiency performance advantages, the back-end optimization process provides a framework that is more amenable to analysis as well.

Multi-sensor fusion localization for autonomous vehicles is mainly based on the GNSS, inertial measurement unit (IMU), camera, LiDAR, and vehicle states [11,12]. LiDAR-based methods can provide precise localization under the condition of weak satellite signals [13]. However, global localization and environmental degradation are still important issues for LiDAR-based methods. Complementary sensor fusion is an effective method to solve these issues. LiDAR shows good performance in scenarios with full 3D or texture features, real time kinematic (RTK) GNSS provides a precise absolute position, and IMU and vehicle states provide the position and orientation of the vehicle getting rid of the external scenarios.

Taking the above-mentioned into consideration, LiDAR-based SLAM and localization still have problems to be solved. A SLAM and localization method based on multi-sensor fusion is proposed and integrated into a general framework in this paper, to solve the map drift and localization failure and meet the demand of high-precision localization under the condition of unavailable satellites, extreme climate, or road structure changes. A pose graph considering the loop closure and RTK-GNSS position is used to optimize the map. The LiDAR-based localization result and vehicle states are integrated into an error state Kalman filter (ESKF) to obtain robust and precise localization.

**Figure 1:**
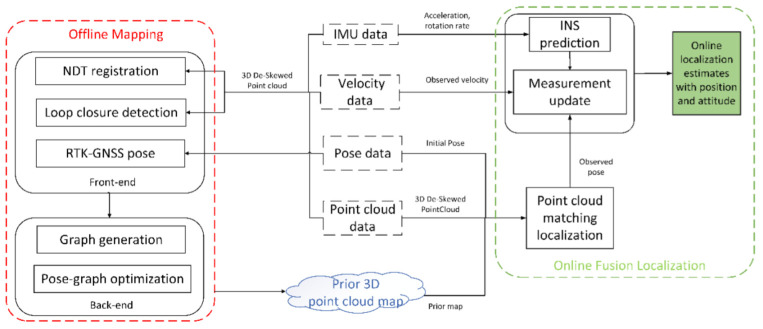
The framework that estimates the optimal position and attitude (PA) for the autonomous vehicle by combining offline mapping and online localization. NDT: normal distributions transform, RTK-GNSS: real time kinematic-global navigation satellite system, IMU: inertial measurement unit, INS: inertial navigation system.

Figure 1 is the framework of this article. In the process of offline mapping, a pose-graph optimization LiDAR SLAM is proposed based on NDT registration, loop closure detection and RTK-GNSS position constraints to generate an optimized 3D point cloud map. In the online localization process, the inertial navigation system (INS) is used as a prediction model in the Kalman filter propagation phase, LiDAR localization and vehicle velocity are used by an error-state Kalman filter as the measurements.

The main contributions of this paper are summarized as follows.

- The NDT registration, scan context-based loop closure detection and RTK-GNSS are integrated into a LiDAR SLAM framework and innovative use of pose graph to combine multiple methods to optimize position and reduce map drift.
- LiDAR matching localization position and vehicle states are fused by ESKF, which takes full advantage of the vehicle velocity constraints of ground autonomous vehicles to optimize localization results and provide robust and accurate localization results.
- A general framework with mapping and localization is proposed, which is tested on the KITTI dataset [14] and real scenarios. Results demonstrate the effectiveness of the proposed framework.

The rest of the paper is structured as follows. The related work about mapping and localization is presented in Section 2. The offline mapping process is introduced in Section 3 and the online localization method introduced in Section 4. The experiment evaluation is given in Section 5. The discussion is given in Section 6. Finally, the conclusion and future work are presented in Section 7.

## 2. Related Work

In this section, a brief overview of algorithms related to LiDAR SLAM and multi-sensor fusion localization methods are introduced, including the point cloud registration algorithms, loop closure detection algorithms, pose graph algorithms, filter-based sensor fusion algorithms, and their interaction.

With the development of LiDAR SLAM, various registration algorithms have been proposed. The ICP algorithm is widely used in the registration of point cloud. Due to the improvement of computational efficiency and accuracy requirements, a variety of variant ICP algorithms have been derived [15]. However, the ICP is very sensitive to the initial guess. Different from the ICP, the NDT registration algorithm builds a statistical probability model of the point cloud, which is more efficient and accurate. Study [16] proposed a 3D-NDT registration algorithm as the improvement of the 2D-NDT algorithm [17] and compares qualitatively and quantitatively with the standard ICP algorithm. Results show that the method is faster and more reliable. Study [7] proposed an NDT-based SLAM method, which can achieve long-range high-precision map establishment and localization in dynamic scenarios. Li et al. [18] improved the accuracy of stereo visual SLAM by removing dynamic obstacles. Wen et al. [19] compared the performance of NDT-based graph optimization SLAM under complex urban conditions; the results show that the performance of the NDT SLAM algorithm is positively related to the traffic environment.

Loop closure is essential for correcting drift error and building a globally consistent map [5]. Visual-based loop closure detection is often limited by illumination variance and environment changes. The early LiDAR-based methods for place recognition focus on descriptors from structural information [20]. However, the descriptor method is limited by rotational invariance and poor point cloud resolution. Study [21] proposed a histogram method to address these problems but still causes false recognition. To address the aforementioned issues, studies [22,23] proposed the scan context method, which proposed a more efficient bin encoding function for place recognition and is widely used in LiDAR SLAM currently; in addition, the loop closure detection method based on deep learning has also been gradually applied to SLAM [24].

Graph-based optimization [25], which optimizes the full trajectory and map of the vehicle from the full set of measurements, has received attention in many studies in recent years. Some general frameworks and open-source implementation of a pose-graph method are proposed by [26,27]. Study [28] proposed a tutorial for the reader to implement graph-based SLAM. To improve the robustness of pose-graph SLAM, study [29] proposed a novel formulation that allows the back-end to change parts of the topological structure of the graph during the optimization process and progress experiments by loop closure constraints. To obtain accurate positions for mapping in large-scale environment, study [30] proposed global positioning system (GPS) and LiDAR odometry (GLO)-SLAM, which uses LiDAR to verify the reliability of GNSS, and the LiDAR odometry also will be optimized by means of reliable GPS data. In addition, study [31] added IMU/odometry pre-integration results under the framework of graph optimization, which effectively reduced navigation drift. With the development of deep learning, related technologies have also been applied to the field of SLAM [32,33].

The multi-sensor fusion method is usually used in SLAM and localization areas. Fusing multiple sensors and making the whole system accurate, robust, and applicable for various scenes is a challenging task. Study [34] integrated 2D LiDAR/IMU/GPS into a localization system for urban and indoor scenarios, IMU and RTK-GNSS for full scene localization, and vehicle velocity is good complementary information for localization, especially in satellites denied and environmental degradation conditions.

## 3. The Offline Mapping

The online LiDAR localization module relies on a pre-build map. The offline mapping aims to obtain a 3D point cloud map representation of the scenario. The NDT-based point cloud registration and scan context-based loop closure detection are innovatively combined into the front-end and the pose-graph is used in the back-end to optimize the map.

### 3.1. LiDAR SLAM Front-End

#### 3.1.1. NDT Based Registration

Comparing with the ICP algorithm, the NDT divides the point cloud space into cells and each cell is continuously modeled as a Gaussian distribution. Taking the better calculation efficiency and registration accuracy of NDT into account, the NDT is chosen as the point cloud registration method. The process of NDT can be expressed as follows.

In the point cloud, point sets *X* and *Y* are the consecutive frames, *X* is the frame at the previous moment, *Y* is the frame at the next moment:

(1) $$X=\left\{x_{1},x_{2},\ldots ,x_{n}\right\}$$

(2) $$Y=\left\{y_{1},y_{2},\ldots ,y_{n}\right\}$$

Assuming that the transformation between *X*, *Y* can be expressed as follow:

(3) $$p={\left[T_{x}\;T_{y}\;T_{z}\;R_{x}\;R_{y}\;R_{z}\right]}^{T}$$

where *T* is the translation vector, *R* is the rotation vector.

The mean of all points in *X* can be expressed as:

(4) $$\mu =\frac{1}{N_{x}}\sum_{i=1}^{N_{x}}x_{i}$$

where *N_x_* is the number of points in the *X*. The covariance of *X* can be expressed as follow:

(5) $$\sum =\frac{1}{N_{x}-1}\sum_{i=1}^{N_{x}}(x_{i}-\mu ){\left(x_{i}-\mu \right)}^{T}$$

Assuming that the transformation *p* makes point *y_i_* transform to *y_i_*′, the transformation process can be expressed as the followed:

(6) $$y_{i}{}^{'}=T(p,y_{i})=Ry_{i}+T$$

After transformation, the point *y_i_*′ is in the same coordinate system as the target point set *X*, and its coincidence degree can be expressed as a Gaussian distribution:

(7) $$\begin{array}{l} f\left(X,\;y_{i}{}^{'}\right)=f\left(X,\;T(p\;,\;y_{i})\right)=\\ \frac{1}{\sqrt{2\pi }\sqrt{\left|\left.\sum \right|\right.}}\exp\left(-\frac{{\left(y_{i}{}^{'}-\mu \right)}^{T}\sum^{-1}(y_{i}{}^{'}-\mu )}{2}\right) \end{array}$$

The joint probability distribution of *Y* and *X* can be expressed as follows:

(8) $$\psi =\prod_{i=1}^{N_{y}}f\left(X,\;T(p\;,\;y_{i})\right)$$

where *N_y_* is the number of points in the *Y.*

The objective function can be expressed as follow:

(9) $$\max\psi =\max\prod_{i=1}^{N_{y}}f\left(X,\;T(p\;,\;y_{i})\right)$$

Therefore, the maximize of the joint probability *ψ* means that the transformation has the highest degree of coincidence and the optimization variables *T* and *R* represent the translation and rotation between two consecutive frames, respectively.

#### 3.1.2. Scan Context Based Loop Closure Detection

Comparing with the feature descriptors of the environment, few studies focus on the structural information to describe scenes. Scan context proposes a non-histogram method of global descriptors, which directly records the 3D structure of the visible space and can be deployed in LiDAR-based place recognition effectively. The lightweight and efficient encoding method, which can improve the accuracy of loop closure detection, is conducive to storing point cloud information. The scan context method is applied for the offline mapping process. Firstly, scan context is used to detect the loop closure frame. After detecting the candidate frame in the historical frame, NDT is used to register the loop closure frame with the current point cloud frame to obtain the precise loop pose.

**Figure 2:**
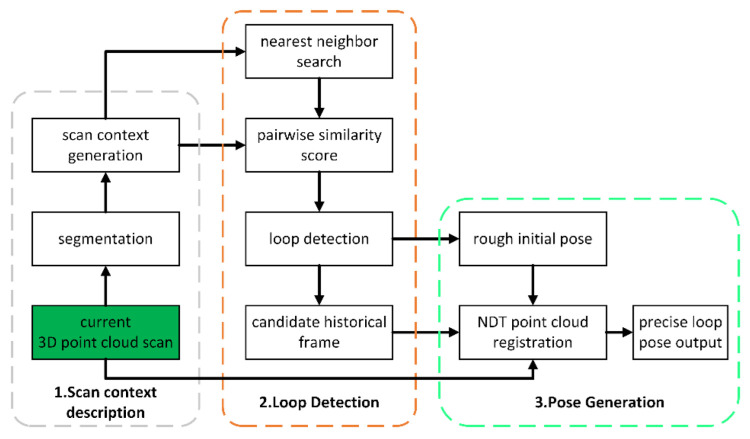
Scan context-based loop closure detection, current 3D point cloud scan is the start of this process. Which can provide loop closure detection position for offline mapping position optimization.

Figure 2 shows the flow chart of scan context and loop closure detection. In the point cloud segmentation process, the point cloud space is cut into *N_r_* rings along the increasing radius and the rings are cut into *N_s_* sectors:

(10) $$d_{r}=\frac{L_{\max}}{N_{r}}$$

where *d_r_* represents the width of the ring, *L_max_* represents the maximum measurement distance of LiDAR, *N_r_* is numbers of rings.

After segmentation, the segmented bin cells can be represented as a set *P*:

(11) $$P=\underset{i\in [N_{r}],j\in [N_{s}]}{\cup }p_{ij}$$

where *p_ij_* represents the set of midpoints of the *i^th^* circle segmentation unit of the *j^th^* sector.

In the generation of scan context process, the scan context *I* is represented as a *N_r_* × *N_s_* matrix, each element in the matrix represents the maximum value of all 3D points in the z-direction.

The distance function between two frames of point cloud scan context is defined as:

(12) $$d(I^{q},I^{c})=\frac{1}{N_{s}}\sum_{j=1}^{N_{s}}\left(1-\frac{c_{j}^{q}\cdot c_{j}^{c}}{\left\|c_{j}^{q}\right\|\left\|c_{j}^{c}\right\|}\right)$$

which can be used for similarity score, where *I^q^* is the current frame scan context, *c^q^_j_* is the *j^th^* of *I^q^*, *I^c^* is the historical frame scan context, and *c^c^_j_* is the *j^th^* of *I^c^*.

To solve the problem that the current frame is rotated relative to the historical frame, the order of the column vectors in the scan context obtained at the current time is changed and causes a large-distance function between the two frames, the historical frame *I^c^* is translated by column, which will obtain *N_s_* scan contexts, calculate the distance from the scan context of the current frame in turn, and select the one with the smallest distance, which can be expressed as follows:

(13) $$D\left(I^{q},I^{c}\right)=\min_{n\in [N_{s}]}d\left(I^{q},I_{n}^{c}\right)$$

The loop frame can be found by comparing the similarity of scan contexts between the current frame and the historical frame point cloud; when the distance function is less than a certain threshold, the historical frame is considered to be a loop frame.

In the precise position calculation process, scan context is used to calculate the rotation angle *φ* between the current frame and the loop frame to improve the calculation efficiency and accuracy of the NDT, and *φ* is used as the initial position for the NDT registration process:

(14) $$n^{*}=\underset{n\in [N_{s}]}{\arg\min d\left(I^{q},I_{n}^{c}\right)}$$

(15) $$\varphi =\frac{2\pi }{N_{s}}\cdot n^{*}$$

#### 3.1.3. RTK-GNSS Based Localization

Real time kinematic localization is a satellite navigation technique used to enhance the precision of localization data derived from satellite-based navigation systems. RTK relies on a single reference station to provide real-time corrections for the rover providing up to centimeter-level accuracy. There are indeed many situations with severe multipath and signal blockage under urban buildings or in forests. A stable and precise position and attitude can be provided for autonomous vehicles by combining RTK-GNSS and IMU.

### 3.2. Back-End Optimization

After interframe association and submap matching, there are inevitable cumulative errors in the point cloud map. The method of pose-graph optimization is used to further eliminate the cumulative errors, and the loop closure position and RTK-GNSS position will be used as constraints, the back-end optimization step is summarized in Algorithm 1.
**Algorithm 1.** The process of back-end optimization**Input:**LiDAR odometry position *x_i_, x_j_*RTK-GNSS position *z_i_*Loop closure position *z_i,j_*′**Output:**Optimized vehicle position *x_opt_* 1: Trajectory alignment for *x_i_, z_i_* and *z_i,j_*′ 2: **for** each position *x_i_* **do** 3:   **if** meet optimization cycle times *h* **then** 4:    execute optimization process: 5:    *x_opt_ = arg min F*(*x_i_, x_j_, z_i_, z_i,j_*′) 6:   **else** **7:    **add RTK-GNSS position *z_i_* constraint 8:    **if** loop closure position detected **then** 9:      add loop closure position *z_i,j_*′ constraint 10:    **end if** 11:   **end if** 12: **end for** 13: **return** optimized vehicle position *x_opt_*

#### 3.2.1. Graph Generation

**Figure 3:**
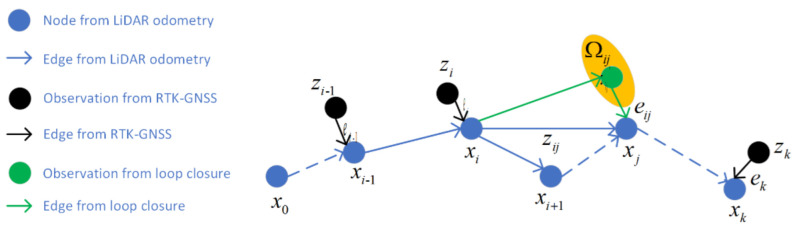
Graph structure for back-end optimization.

The graph consists of edges and nodes, as shown in Figure 3; *x_i_* represents nodes from LiDAR odometry, *z_i_* represents prior position from RTK-GNSS, *e_i_* represents the edge between *x_i_* and *z_i_*. *z_ij_* represents the transformation of *x_j_* and *x_i_*, *z_ij_*′ represents expected observation from loop closure, and *e_ij_* represents the edge between *z_ij_* and *z_ij_*′.

#### 3.2.2. Graph Optimization

Graph optimization takes all the constraints into a non-linear optimization problem, which will consider all the observation measurements:

(16) $$F(x)={\sum_{i,j}e(x_{i},x_{j},{\overset{\wedge }{z}}_{ij})}^{T}\Omega_{ij}e(x_{i},x_{j},{\overset{\wedge }{z}}_{ij})$$

where *F*(*x*) represents errors between all edges. Ω*_ij_* is the matrix indicating the importance of each constraint in the global graph optimization. To adjust the state quantity *x* to minimize the value of the residual, it is necessary to obtain the Jacobian of the residual relative to state quantity, and then use the gradient descent method to optimize. The solution of this optimization is the *x_opt_* which satisfying the following function:

(17) $$x_{opt}=\arg\min F(x)$$

To integrate the RTK-GNSS into the graph optimization, the error between LiDAR odometry *x_i_* and RTK-GNSS position *z_i_* can be represented as follows:

(18) $$e_{i}=\ln{\left(z_{i}{}^{-1}x_{i}\right)}^{\vee }$$

The residual *e_i_* after adding disturbance term to the *x_i_* can be expressed as follows:

(19) $$\overset{\wedge }{e_{i}}=\ln{\left(z_{i}{}^{-1}\exp(\delta \xi_{i}{}^{\wedge })x_{i}\right)}^{\vee }$$

The error between *z_ij_* and *z_ij_*′ can be represented as follows:

(20) $$e_{ij}=\ln{\left(z_{ij}{}^{-1}x_{i}{}^{-1}x_{j}\right)}^{\vee }$$

The residual *e_ij_* after adding disturbance to the *x_i_* and *x_j_* can be expressed as follows:

(21) $$\overset{\wedge }{e_{ij}}=\ln{\left(z_{ij}{}^{-1}x_{i}{}^{-1}\exp({\left(-\delta \xi_{i}\right)}^{\wedge })\exp(\delta \xi_{j}{}^{\wedge })x_{j}\right)}^{\vee }$$

The residual is expanded after adding disturbance term, and the Jacobian matrix *J* of the residual with respect to the state quantity can be obtained.

A first-order taylor expansion on the residuals can be expressed as follows:

(22) e(x+Δx)≈e+JΔx

(23) $$F(x+\Delta x)\approx {\left(e+J\Delta x\right)}^{T}\Omega_{ij}(e+J\Delta x)$$

The state quantity *x_opt_* after correction can be expressed as follows:

(24) $$x_{opt}=x+\Delta x$$

## 4. The Online Localization

A multi-sensor fusion localization method based on the ESKF is proposed, which will estimate the vehicle position and attitude (PA) jointly by fusing vehicle states and LiDAR localization pose.

### 4.1. LiDAR Localization Based on 3D Point Cloud Map

The LiDAR localization based on a 3D point cloud map estimates the position of the vehicle in real-time, and the position can be used for the Kalman filter observation update. In this process, the RTK-GNSS position is used as the initial position for LiDAR localization to improve matching accuracy and efficiency. The NDT algorithm is used as registration method to match the real-time point cloud with the local map, the LiDAR localization step is summarized in Algorithm 2.
**Algorithm 2.** LiDAR localization in prior 3D point cloud map**Input:**RTK-GNSS position *z_i_*Point cloud *p_i_*Prior 3D point cloud global map *M***Output:**LiDAR localization position *x_lidar_* 1: Load 3D point cloud map *M* 2: **if** get the initial position *z_i_* **then** 3:   load local submap *M_sub_* from global map *M* 4:   **if** need update submap *M_sub_* **then** 5:    update submap *M_sub_* 6:   **else** 7:    calculate position between *p_i_* and *M_sub_*: 8:     NDT registration *x_lidar =_ p_i_ ∝ M_sub_* 9:   **end if** 10: **else** 11:   wait for initial position *z_i_* 12: **end if** 13: **return** LiDAR localization position *x_lidar_*


### 4.2. Filter State Equation

In the filter, the state variables error is expressed as follows:

(25) $$X={\left[\delta P^{T},\delta V^{T},\varphi^{T},\varepsilon^{T},\nabla^{T}\right]}^{T}$$

where *δP* is the position error, *δV* is the velocity error, *ϕ* is the attitude error, *ε* is the gyroscope bias error, and ∇ is the accelerometer bias error. The state transition equation in continuous time can be expressed as follows:

(26) $$X=F_{t}X+B_{t}W$$

According to the derivation of the IMU kinematics model, where

(27) $$F_{t}=\left[\begin{array}{ccccc} 0_{3X3}\; & I_{3X3}\; & 0_{3X3}\; & 0_{3X3}\; & 0_{3X3}\\ 0_{3X3}\; & 0_{3X3}\; & F_{23}\; & 0_{3X3}\; & C_{b}^{n}\\ 0_{3X3}\; & 0_{3X3} & F_{33} & C_{b}^{n} & 0_{3X3}\\ \; & \; & 0_{3X15}\; & \; & \\ \; & \; & 0_{3X15}\; & \; & \end{array}\right]$$

(28) $$F_{23}=\left[\begin{array}{lll} 0\; & -f_{U}\; & -f_{N}\;\\ f_{U}\; & 0\; & -f_{E}\;\\ -f_{N}\; & f_{E}\; & 0 \end{array}\right]$$

The east-north-up (ENU) and right-forward-up (RFU) are chosen as the navigation reference frame *n*, and the body frame *b,* respectively, where *f_E_* is the acceleration in the east direction, *f_N_* is the acceleration in the north direction, *f_U_* is the acceleration in the up direction, and *C^n^_b_* is the direction cosine matrix from *b* frame to *n* frame:

(29) $$F_{33}=\left[\begin{array}{ccc} 0\; & w\sin L\; & -w\cos L\;\\ -w\sin L\; & 0\; & 0\;\\ w\cos L\; & 0\; & 0\; \end{array}\right]$$

where *ω* is the angular velocity of the earth’s rotation, *L* is the latitude, *W* includes the gyroscope noise *ω_g_* and accelerometer noise ω*_a_*:

(30) $$W={\left[w_{gx}\;w_{gy}\;w_{gz}\;w_{ax}\;w_{ay}\;w_{az}\right]}^{T}$$

(31) $$B_{t}=\left[\begin{array}{ll} 0_{3X3}\; & 0_{3X3}\\ 0_{3X3}\; & C_{b}^{n}\\ -C_{b}^{n}\; & 0_{3X3}\\ 0_{6X3}\; & 0_{6X3} \end{array}\right]$$

### 4.3. Filter Measurement Update Equation

To compensate the loss of localization signal under complex driving scenarios and enhance the robustness of the localization system, LiDAR localization position and vehicle velocity are used as observation inputs:

(32) $$Y=\left[\delta P_{L}^{T}\;\varphi_{L}^{T}\;\delta V_{v}^{T}\right]$$

where *δP_L_* is the LiDAR localization position error, *φ_L_* is the attitude error, and *δV_v_* is the vehicle velocity error.

The observation equation is as follows:

(33) $$Y=G_{t}X+C_{t}N$$

where

(34) $$G_{t}=\left[\begin{array}{l} I_{3X3}\;0_{3X3}\;0_{3X3}\;0_{3X6}\\ 0_{3X3}\;0_{3X3}\;I_{3X3}\;0_{3X6}\\ 0_{3X3}\;I_{3X3}\;0_{3X3}\;0_{3X6} \end{array}\right]$$

*N* is the observation noise and can be expressed as follows:

(35) $$N={\left[n_{P_{L}^{E}}\;n_{P_{L}^{N}}\;n_{P_{L}^{U}}\;n_{\varphi_{L}^{E}}\;n_{\varphi_{L}^{N}}\;n_{\varphi_{L}^{U}}\;n_{V_{v}^{E}}\;n_{V_{v}^{N}}\;n_{V_{v}^{U}}\right]}^{T}$$

(36) $$C_{t}=\left[\begin{array}{l} I_{3X3}\;0_{3X3}\;0_{3X3}\;\\ 0_{3X3}\;I_{3X3}\;0_{3X3}\;\\ 0_{3X3}\;0_{3X3}\;I_{3X3} \end{array}\right]$$

## 5. Experimental Verification and Performance Analysis

This section introduces experiments with the KITTI dataset and field tests based on the proposed method.

### 5.1. The Experiment Based on KITTI Dataset

**Figure 4:**
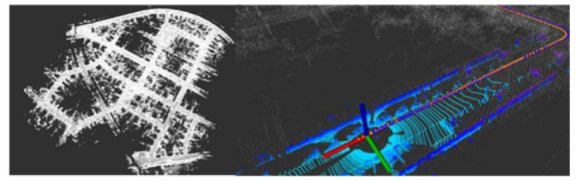
Optimized map and LiDAR localization result based on KITTI dataset.

The KITTI dataset was jointly founded by the Karlsruhe Institute of Technology in Germany and the Toyota American Institute of Technology. It is currently the world’s largest autonomous driving localization and computer vision algorithm evaluation dataset. It contains LiDAR data, IMU data, RTK-GNSS data, velocity data, and the localization groundtruth, which is used to evaluate the mapping and localization accuracy. KITTI has multiple sequence datasets for various scenarios; sequence 00 was used in this study. The mapping and localization result is shown in Figure 4.

#### 5.1.1. Mapping Performance Analysis Based on KITTI Dataset

**Figure 5:**
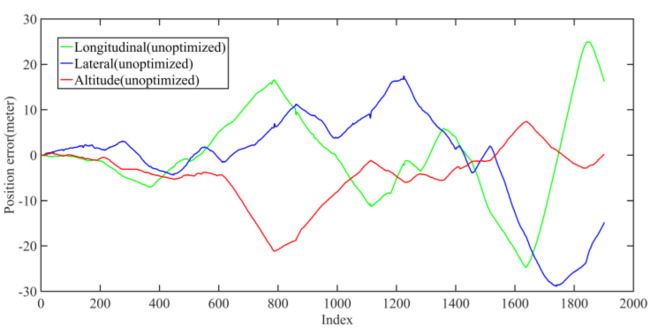
The unoptimized position error in longitudinal, lateral, and altitude directions of KITTI dataset, respectively.

**Figure 6:**
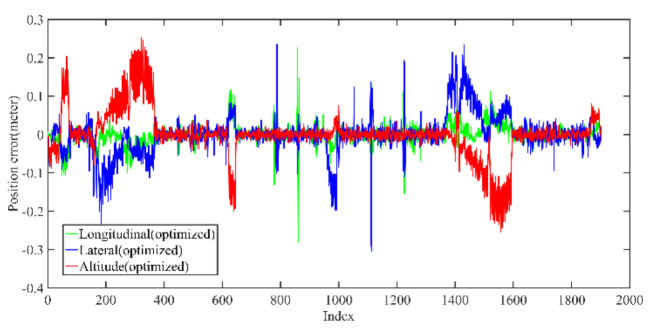
The optimized position error in longitudinal, lateral, and altitude directions of KITTI dataset, respectively; the error is significantly reduced after optimization.

Back-end optimization plays an important role in the process of mapping; Figure 5 and Figure 6 show the results of optimization performance, the abscissa in the figure represents the index of the data frame where the position is saved; in the following, the abscissa is represented by Index, which has the same meaning. In Figure 6, it can be seen that the optimized longitudinal, lateral and altitude error are reduced to centimeter-level, which effectively eliminates the cumulative error of the front-end odometry and improves the mapping accuracy.

**Figure 7:**
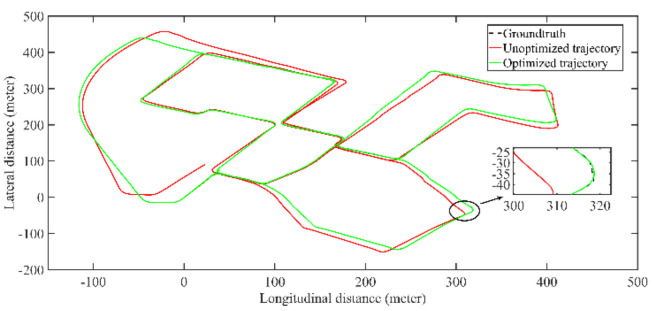
The comparison of trajectory before and after optimization with groundtruth.

Figure 7 shows the coincidence degree between the groundtruth estimated trajectory. It can be seen that there is a large deviation between the unoptimized trajectory and the groundtruth, the optimized trajectory error is significantly reduced. Table 1 shows the trajectory accuracy after optimization is significantly improved and basically coincides with the groundtruth, and the average error is about 10 cm.

#### 5.1.2. Localization Performance Analysis Based on KITTI Dataset

**Figure 8:**
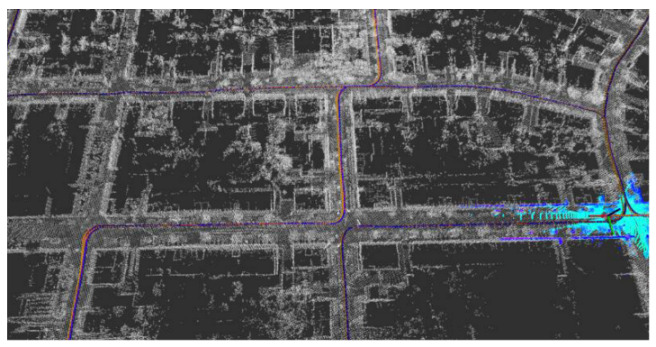
LiDAR localization based on prior KITTI point cloud map; the white points in the figure are the point cloud map built by the offline mapping process, the red line is the trajectory of LiDAR matching results, the orange line is groundtruth, and the blue line is the fused trajectory.

**Figure 9:**
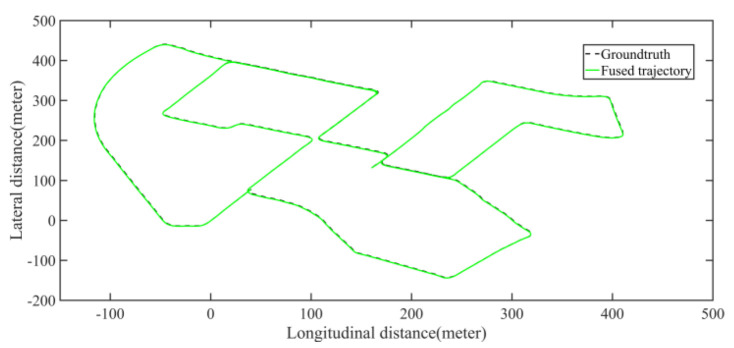
The comparison of fused trajectory and groundtruth. The fused localization data basically coincides with the groundtruth, indicating that the online localization results meet expectations.

The LiDAR localization result is fused with IMU and vehicle velocity to improve the localization accuracy in the case of scenario degradation. As shown in Figure 8 and Figure 9, a localization performance test is conducted based on the prior KITTI point cloud map.

**Figure 10:**
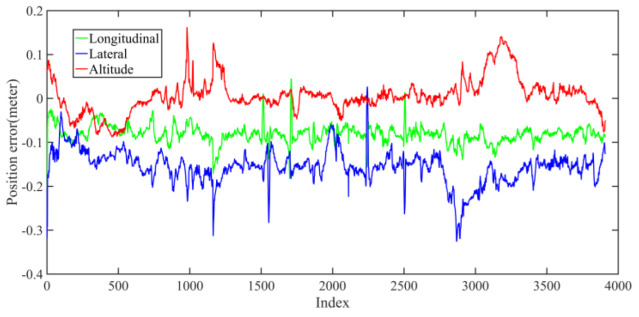
The position error of localization result in longitudinal, lateral, and altitude directions, respectively.

As shown in Figure 10 and Table 2, the maximum position error on the KITTI dataset is within 35 cm, the average position error is within 20 cm, and a stable localization result is maintained.

### 5.2. The Field Test Vehicle and Test Results

To further verify the effectiveness of the proposed method, a four-wheel steering and four-wheel hub motor drive vehicle is developed by our team. It is equipped with sensors for data collection and can feedback vehicle states information through the controller area network (CAN) bus.

#### 5.2.1. Test Vehicle and Sensor Configuration

**Figure 11:**
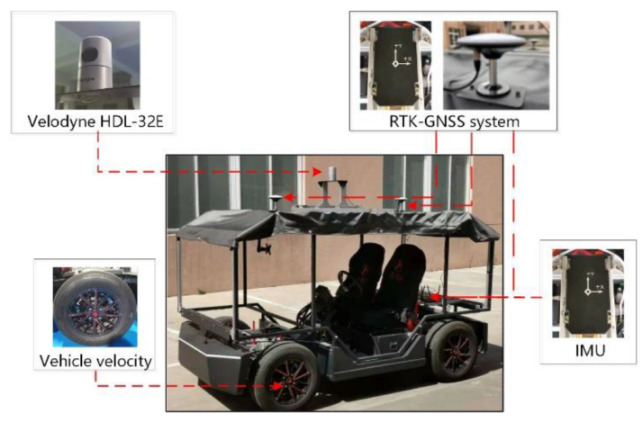
Test vehicle and sensors configuration.

A 32 beams LiDAR, an RTK-GNSS system and an IMU are equipped on the testing intelligent electric vehicle. Sensor specifications of the test vehicle are shown in Figure 11 and Table 3. Gyro and accelerometer bias stability of IMU are 5 deg/h and 0.5 mg, respectively. In addition, the vehicle velocity can be obtained from the on-board CAN bus, and the groundtruth is provided by RTK-GNSS system.

#### 5.2.2. Field Test Mapping Performance Analysis

The proposed mapping method was tested in a real scenario to verify its performance. In the field test, the dataset was collected in the industrial park with scenario change. 

**Figure 12:**
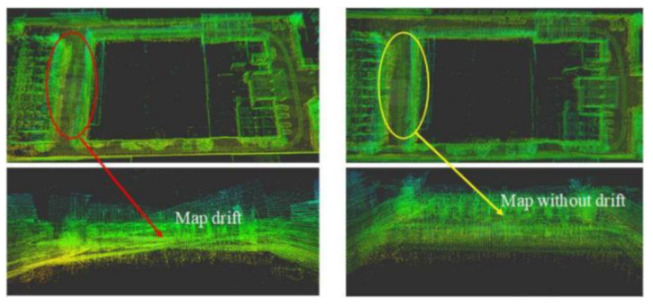
The map drift in small scale scenario is eliminated by back-end optimization. The figure on the left is unoptimized, and the right one is optimized. It can be seen that the map drift of the optimized map is significantly reduced.

**Figure 13:**
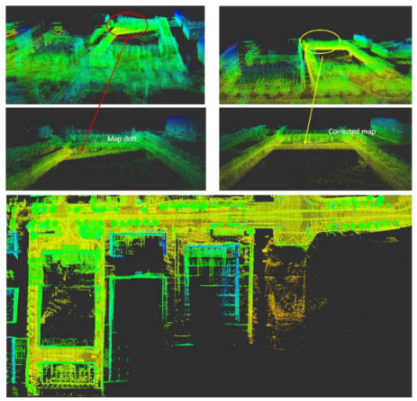
The map drift in large scale scenario is eliminated by back-end optimization. The figure on the upper left is unoptimized, and the upper right one is optimized. It can be seen that the map drift of the optimized map is significantly reduced. The bottom figure is the complete large-scale map after optimization.

**Figure 14:**
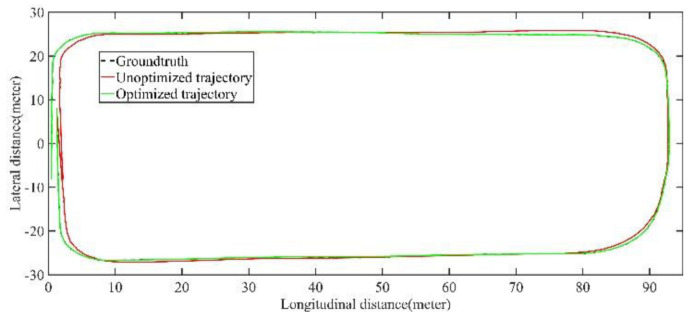
The comparison of trajectory before and after optimization with groundtruth of small scale scenario.

**Figure 15:**
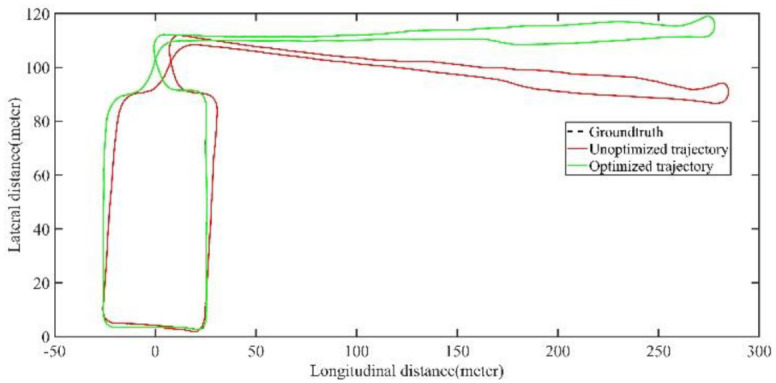
The comparison of trajectory before and after optimization with groundtruth of large scale scenario.

**Figure 16:**
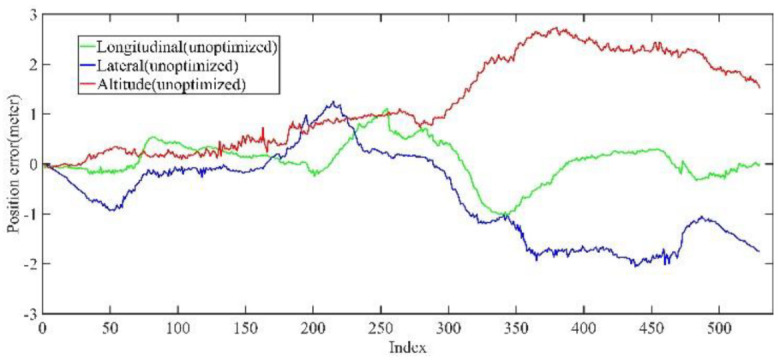
Small scale scenario unoptimized mapping trajectory position error in longitudinal, lateral, and altitude directions. Before optimization, the error is at the meter level.

**Figure 17:**
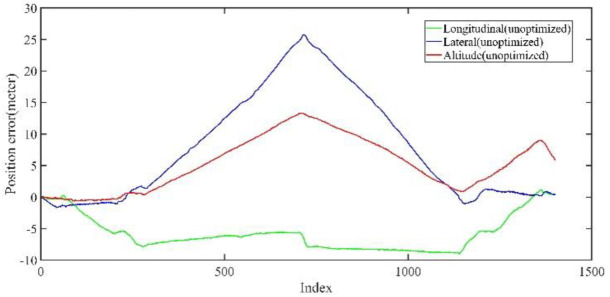
Large scale scenario unoptimized mapping trajectory position error in longitudinal, lateral, and altitude directions. Before optimization, the error is at the meter level.

**Figure 18:**
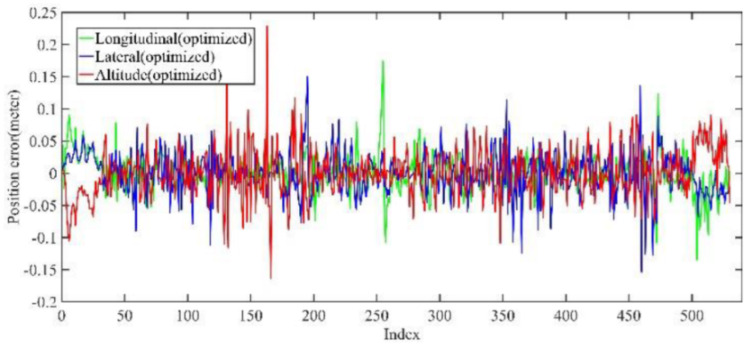
Small scale scenario optimized mapping trajectory position error in longitudinal, lateral, and altitude directions. After optimization, the error is at the centimeter level.

**Figure 19:**
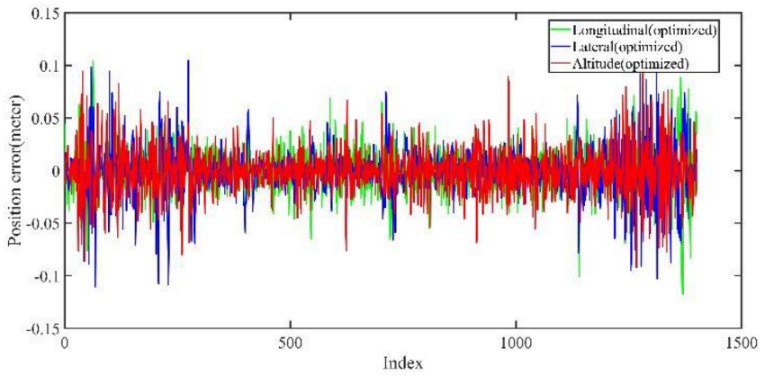
Large scale scenario optimized mapping trajectory position error in longitudinal, lateral, and altitude directions. After optimization, the error is at the centimeter level.

As shown in Figure 12 and Figure 13, two point cloud maps were constructed by offline mapping process, and the map drift was effectively eliminated after optimization. As shown in Figure 14 and Figure 15, due to the use of graph optimization algorithm, the optimized trajectory basically coincides with the groundtruth. It can be seen from Figure 16 and Figure 17 that the position error of the three axes before optimization gradually increases over 1 m, and the average error before the optimization is 1.6 m. As shown in Figure 18 and Figure 19, the error of the three axes is reduced to centimeter-level after optimization, and the average error is 8 cm. It can be seen from the above analysis that the proposed mapping method can reduce the position error significantly and construct a globally consistent map.

#### 5.2.3. Field Test Localization Performance Analysis

Based on the prior point cloud map, five different field tests were implemented to verify the localization performance. The field tests included different driving conditions and scenarios.

**Figure 20:**
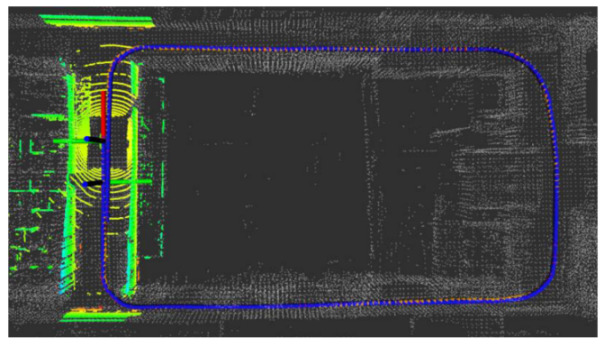
Field test NO.1. One lap under normal driving scenario localization test in prior point cloud map. The white points in the figure are the point cloud map built by the offline mapping process, the red line is the trajectory of LiDAR matching results, the orange line is groundtruth, and the blue line is the fused trajectory.

**Figure 21:**
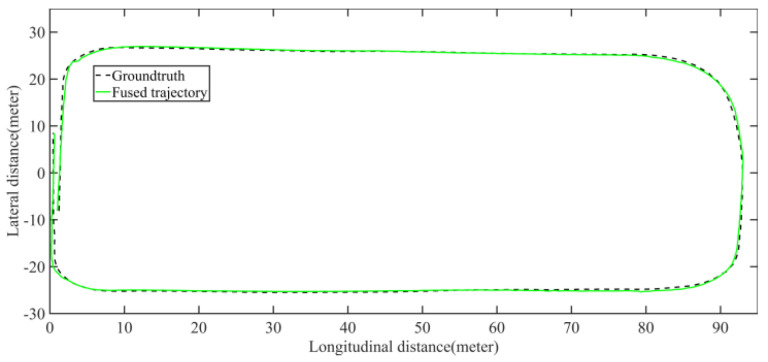
Field test NO.1. One lap under normal driving scenario localization test trajectory comparison. The comparison of fused trajectory with groundtruth.

**Figure 22:**
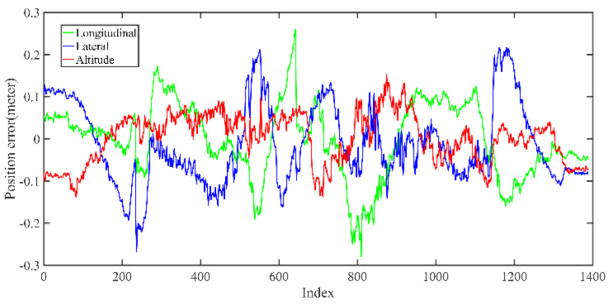
Field test NO.1. One lap under normal driving scenario localization error in longitudinal, lateral, and altitude directions, respectively; the localization error is at the centimeter level.

**Figure 23:**
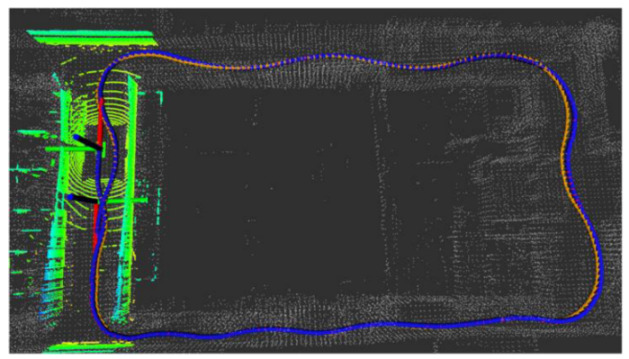
Field test NO.2. One lap under curve driving scenario localization test in prior point cloud map. The white points in the figure are the point cloud map built by the offline mapping process, the red line is the trajectory of LiDAR matching results, the orange line is groundtruth, and the blue line is the fused trajectory.

**Figure 24:**
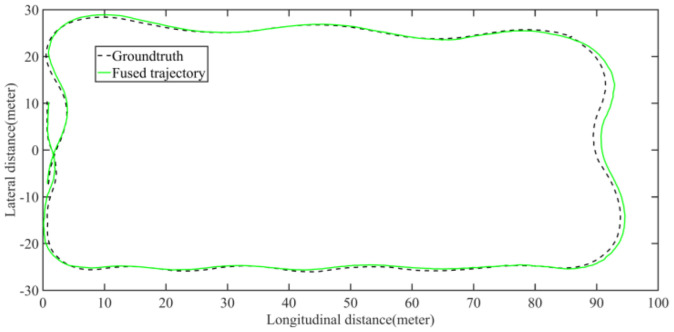
Field test NO.2. One lap under curve driving scenario localization test trajectory comparison. The comparison of fused trajectory with groundtruth.

**Figure 25:**
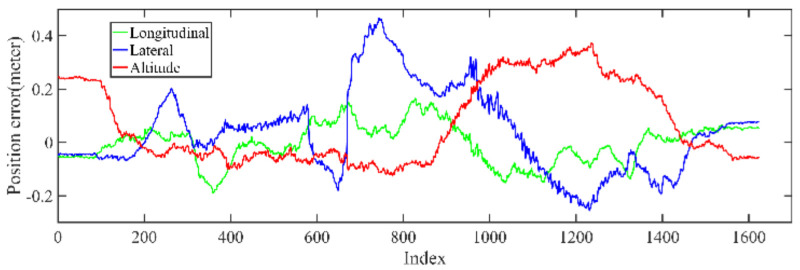
Field test NO.2. One lap under curve driving scenario localization error in longitudinal, lateral, and altitude directions, respectively; the localization error is at the centimeter level.

**Figure 26:**
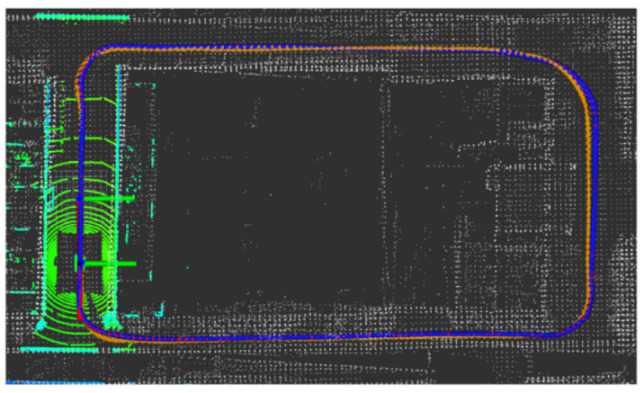
Field test NO.3. Two laps under normal driving scenario localization test in prior point cloud map. The white points in the figure are the point cloud map built by the offline mapping process, the red line is the trajectory of LiDAR matching results, the orange line is groundtruth, and the blue line is the fused trajectory.

**Figure 27:**
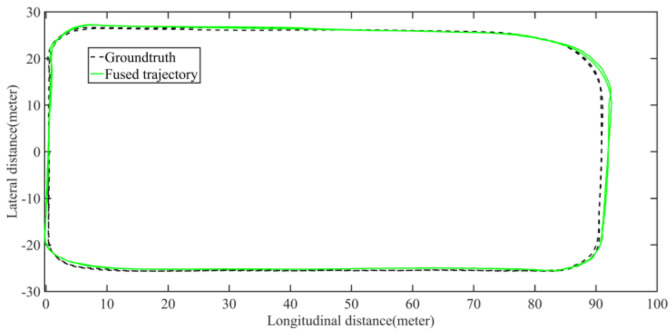
Field test NO.3. Two laps under normal driving scenario localization test trajectory comparison. The comparison of fused trajectory with groundtruth.

**Figure 28:**
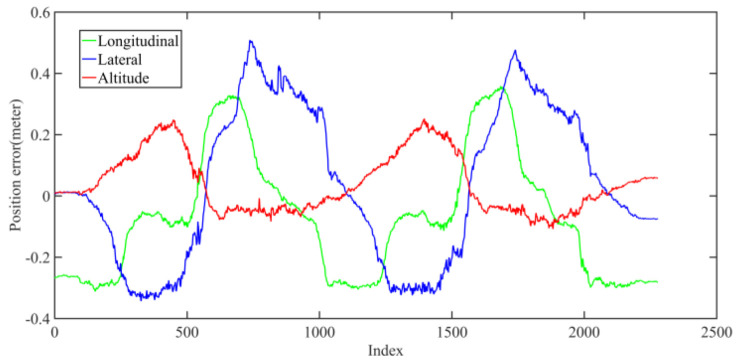
Field test NO.3. Two laps under normal driving scenario localization error in longitudinal, lateral, and altitude directions, respectively; the localization error is at the centimeter level.

**Figure 29:**
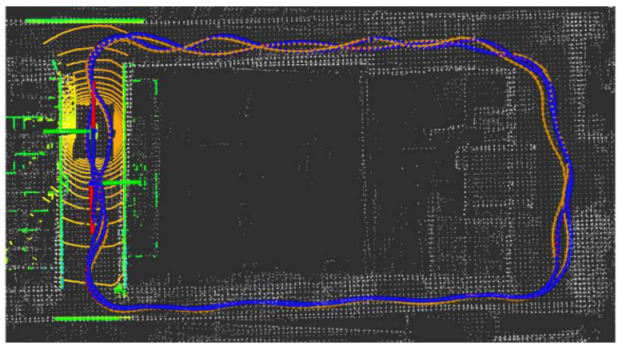
Field test NO.4. Two laps under curve driving scenario localization test in prior point cloud map. The white points in the figure are the point cloud map built by the offline mapping process, the red line is the trajectory of LiDAR matching results, the orange line is groundtruth, and the blue line is the fused trajectory.

**Figure 30:**
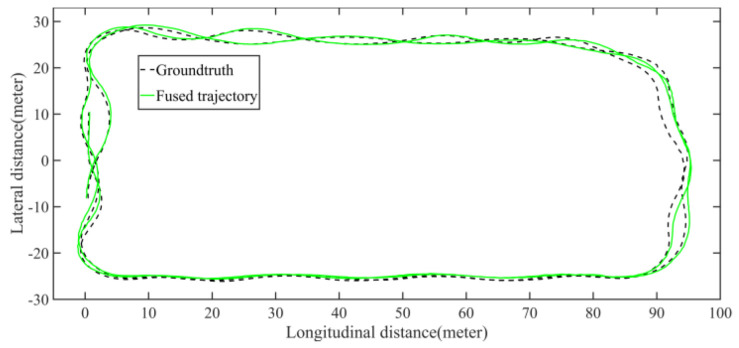
Field test NO.4. Two laps under curve driving scenario localization test trajectory comparison. The comparison of fused trajectory with groundtruth.

**Figure 31:**
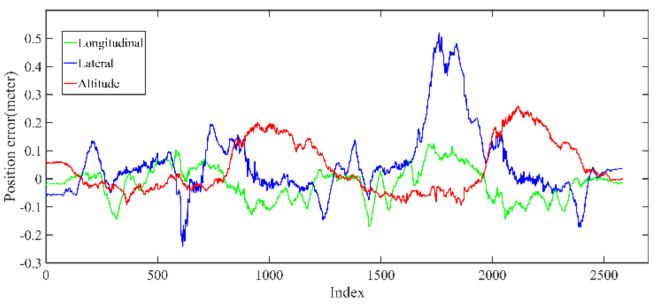
Field test NO.4. Two laps under curve driving scenario localization error in longitudinal, lateral, and altitude directions, respectively; the localization error is at the centimeter level.

**Figure 32:**
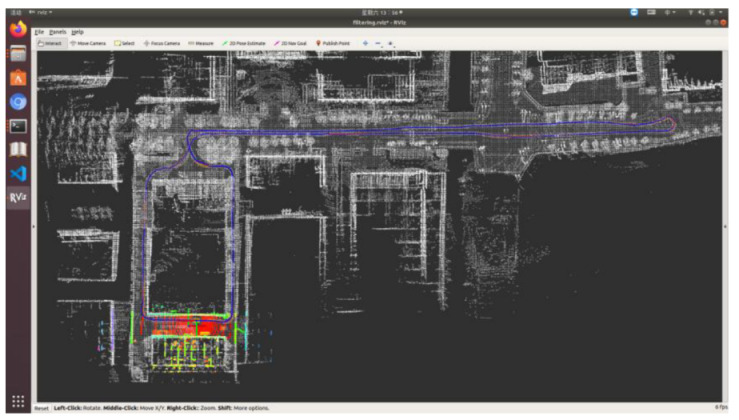
Field test NO.5. Large-scale scenario localization test in prior point cloud map. The white points in the figure are the point cloud map built by the offline mapping process, the red line is the trajectory of LiDAR matching results, the orange line is groundtruth, and the blue line is the fused trajectory.

**Figure 33:**
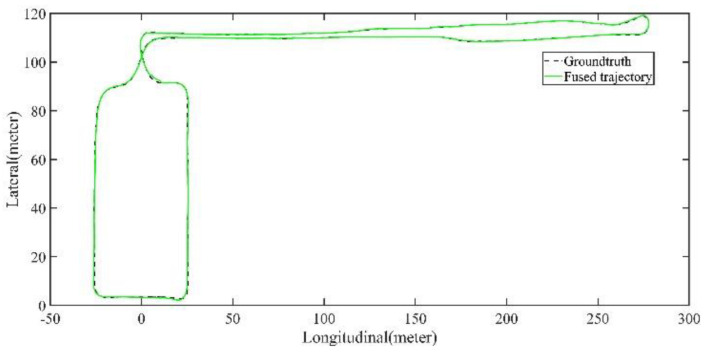
Field test NO.5. Large scale scenario localization test trajectory comparison. The comparison of fused trajectory with groundtruth.

**Figure 34:**
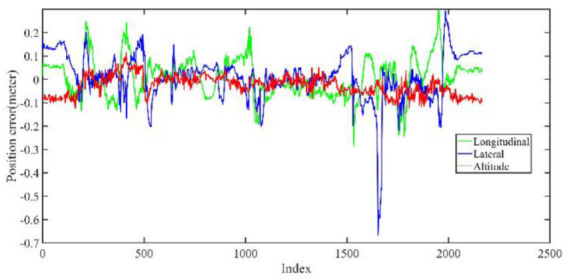
Field test NO.5. Large scale scenario localization error in longitudinal, lateral, and altitude directions, respectively; the localization error is at the centimeter level.

Five sets of field tests represent different driving conditions and travel distances. As shown in Figure 20, Figure 21, Figure 22, Figure 23, Figure 24 and Figure 25, due to the small changes in the scenario, the fused trajectory basically coincides with the groundtruth under the normal driving scenario and the curve driving scenario of one lap, and the maximum error does not exceed 45 cm. From Figure 26, Figure 27, Figure 28, Figure 29, Figure 30 and Figure 31, we conducted another two sets of experiments under different scenarios, due to changes in the environment, the LiDAR localization position has drifted, but the maximum positioning error after fusing the IMU and the vehicle velocity can still be controlled within 55 cm. From Figure 32, Figure 33 and Figure 34, in the large-scale scenario, the localization performance is still robust. It can be seen from Table 4 that the average position error is within 30 cm, which meets the autonomous driving lane-level localization requirements. Field tests scenarios results show that the localization algorithm based on the prior point cloud map can achieve good performance.

## 6. Discussion

From the experimental results, this can all be summarized as the following:

The offline mapping method proposed in this paper can effectively eliminate map drift, provide a mapping accuracy of 5–10 cm, and can be used in localization work to provide a stable and reliable map data source.

In the online localization process, we use the multi-sensor fusion method to achieve a positioning accuracy of 20–30 cm, which benefits from the good mapping accuracy and the design of the multi-sensor fusion model.

## 7. Conclusions and Future Work

This paper presented a LiDAR-based sensor fusion SLAM and localization method for autonomous vehicle offline mapping and online localization. In the mapping process, NDT registration, scan context loop closure detection and RTK-GNSS position are considered in front-end and back-end, innovative use of the pose graph to combine multiple methods to optimize position and reduce map drift, which realizes 5–10 cm mapping accuracy, and the map drift is eliminated effectively. In the online localization process, the ESKF is used to fuse complementary sensor information, such as LiDAR, IMU and vehicle velocity to achieve good localization accuracy in various challenging scenarios, which takes full advantage of the vehicle velocity constraints of ground autonomous vehicles to optimize localization results, and reaches 20–30 cm localization accuracy and shows environmental robustness. Such good and stable mapping and localization results can assist autonomous vehicles to safely complete navigation tasks in the lane. Furthermore, the proposed method can be used to fuse more sensors for offline mapping and online localization, respectively, facing different applications. In the future work, we will try to tightly couple the IMU and LiDAR to the mapping system, which can reduce the drift of the front-end, and improve the quality of the mapping.

## Figures and Tables

**Table 1:** Mapping performance comparison before and after optimization [35] (units: meter).

	Max	Min	Mean	RMSE	STD
Before optimization	34.31	0.02	13.61	16.61	9.53
After optimization	0.23	0.01	0.11	0.13	0.09

**Table 2:** Localization error compared with groundtruth (units: meter).

Max	Min	Mean	RMSE	STD
0.35	0.08	0.18	0.16	0.07

**Table 3:** Sensor specifications of test vehicle (Velodyne HDL-32E from Velodyne, San Jose, CA, USA. StarNeto, Newton-M2 from StarNeto Technology, Beijing, China).

Sensors	Specifications	No.	Frequency/Hz	Accuracy
3D LiDAR	Velodyne, HDL-32E, 32 beams	1	10	2 cm, 0.09 deg
RTK-GNSS system	StarNeto, Newton-M2, L1/L2 RTK	1	50	2 cm, 0.1 deg
IMU	Newton-M2	1	100	5 deg/h, 0.5 mg
Vehicle velocity	On-board CAN bus	1	100	0.1 m/s

**Table 4:** Average errors from 5 field test scenarios.

Field Test Sequence	Test Scenario	Average Error	RMSE
01	Normal driving, 1 lap	21.6 cm	23.2 cm
02	Curve driving, 1 lap	22.5 cm	24.3 cm
03	Normal driving, 2 laps	28.3 cm	29.2 cm
04	Curve driving, 2 laps	25.5 cm	27.1 cm
05	Large scale, 1 laps	29.2 cm	29.6 cm

## Data Availability

Not applicable.
